# Patient-derived organoids (PDOs) as a novel in vitro model for neuroblastoma tumours

**DOI:** 10.1186/s12885-019-6149-4

**Published:** 2019-10-21

**Authors:** P. Fusco, B. Parisatto, E. Rampazzo, L. Persano, C. Frasson, A. Di Meglio, A. Leslz, L. Santoro, B. Cafferata, A. Zin, E. Cimetta, G. Basso, M. R. Esposito, G. P. Tonini

**Affiliations:** 1 Fondazione Istituto di Ricerca Pediatrica Città della Speranza (IRP) - Neuroblastoma Laboratory Corso Stati Uniti 4, 35127 Padova, Italy; 2 Fondazione Istituto di Ricerca Pediatrica Città della Speranza (IRP) – Corso Stati Uniti 4, 35127 Padova, Italy; 3 University of Padova, Department of Women’s and Children’s Health, 35128 Padova, Italy; 4 Department of Medicine DIMED, Pathology and Cytopathology Unit, University of Padua, 35127 Padova, Italy; 5 University of Padua, Department of Industrial Engineering (DII), 35127 Padova, Italy

**Keywords:** Neuroblastoma, Patient-derived organoids, Preclinical model

## Abstract

**Background:**

Neuroblastoma (NB) is a paediatric tumour of the sympathetic nervous system. Half of all cases are defined high-risk with an overall survival less than 40% at 5 years from diagnosis. The lack of in vitro models able to recapitulate the intrinsic heterogeneity of primary NB tumours has hindered progress in understanding disease pathogenesis and therapy response.

**Methods:**

Here we describe the establishment of 6 patient-derived organoids (PDOs) from cells of NB tumour biopsies capable of self-organising in a structure resembling the tissue of origin.

**Results:**

PDOs recapitulate the histological architecture typical of the NB tumour. Moreover, PDOs expressed NB specific markers such as neural cell adhesion molecules, NB84 antigen, synaptophysin (SYP), chromogranin A (CHGA) and neural cell adhesion molecule NCAM (CD56). Analyses of whole genome genotyping array revealed that PDOs maintained patient-specific chromosomal aberrations such as *MYCN* amplification, deletion of 1p and gain of chromosome 17q. Furthermore, the PDOs showed stemness features and retained cellular heterogeneity reflecting the high heterogeneity of NB tumours.

**Conclusions:**

We were able to create a novel preclinical model for NB exhibiting self-renewal property and allowing to obtain a reservoir of NB patients’ biological material useful for the study of NB molecular pathogenesis and to test drugs for personalised treatments.

## Background

Neuroblastoma (NB) is a paediatric cancer originating from neural crest cells with heterogeneous biological, morphological, genetic and clinical characteristics [1] [2]. More than half of the children are diagnosed as a metastatic disease (stage M patients), usually involving the bone marrow and/or skeleton [3]. These patients, classified high-risk (HR)-NB, do not respond to standard therapeutic regimens and relapse with an overall survival (OS) rate lower than 40% at 5 years [4], despite advances in treatment strategies [5]. To date, paediatric oncologists are seeking ways to address treatment based on the NB tumour mutational profile. However, gene sequencing resulted in few actionable mutations [6] and very few drugs can be validated clinically. Thus, the HR-NB patients’ outcome remains grim, making mandatory a wide comprehensive molecular characterisation of tumour biopsies for personalised therapy and to perform preclinical tests addressing drugs activity on the tumour cells. Unfortunately, a limit for this activity is very often the scarcity of biopsy material surgically obtained by HR-NBs patients. Standard culture models are widely exploited for experimentation in cancer research, but the genetic instability of cells leads to a loss in the features of the tumour of origin [7]. Similarly, patient-derived tumour xenografts (PDX), recapitulating the histopathological hallmarks and molecular landscape of the tumour [8] [9], have drawbacks related to the high variability in the time needed for tumour engraftment and the high numbers of tumour cells required.

In this study, we developed the first patient-derived organoids (PDOs) model of NB as representative preclinical in vitro tool able to recapitulate molecular and phenotypic landscape of the original NB tumour. We established PDOs from primary cells tumour biopsy of HR-NB patients stage M. In particular, we demonstrated that PDOs retained the histologic and genomic features of the NB tumours, and preserved the intra-tumour heterogeneity of NB tissue. PDOs can be exploited as clinically relevant models for basic research, to model patient-specific pathology, but also for drug development and to identify the best chemotherapy combination for each patient. However, further studies are needed to assess the PDOs’ wherewithal for several applications. To our knowledge, this is the first organoid model derived from NB primary cells and represents a physiological model exploitable for the screening of personalised effective drugs against NB.

## Methods

### NB-PDOs establishment

PDOs were established from 4 NB patients stage M (HR-NB with metastatic disease) (N691, N700, N711, N772) cells kindly provided by J. Molenaar (Academic Medical Centre, University of Amsterdam). Tumour biopsies were handled according to procedures reported by Bate-Eya et al. [10] to obtain single-cells and for their molecular and genomic characterization. The tumour sample was classified as NB Schwannian stroma-poor according to the International NB Pathology Committee [3] and contained more than 60% of neuroblasts in tumour cells. To initiate PDOs culture, we modified the procedure reported by Hubert CG et al., 2016 [11]. Cells suspension was embedded in Matrigel (5,250,005, Sacco S.r.l., Como, Italy) and 20 μl droplets were placed on parafilm molds and incubated for 1 h at 37 °C. Then, droplets were transferred in 12-well plates and cultured in DMEM-F12 (Aurogene, Rome, Italy) supplemented with 1% Glutamine (Life Technologies, Carlsbad, CA, USA), 1% Penicillin/Streptomycin antibiotics (Life Technologies, Carlsbad, CA, USA), 40 ng/ml basic fibroblast growth factor (bFGF) (Sigma-Aldrich, Missouri, USA), 1% B27 (Gibco, USA), 20 ng/ml epidermal growth factor (EGF) (Cell Guidance System Ltd., Cambridge, UK), 1% N2 (ThermoFisher Scientific, Massachusetts, USA), 10% BIT 9500 Serum Substitute (STEMCELL Technologies, Canada Inc.), 5% MEM non essential amino acids (Biowest, Nauillé, France), 55 μM β-mercaptoethanol (Sigma Aldrich, Missouri, USA) and 1000 U/ml leukaemia inhibitory factor (LIF) (Voden medical instruments, Monza-Brianza, Italy). PDOs were cultured for 40 days before characterisation. Images of growing organoids were acquired using DeltaPix DP 200 program (Exacta-Optech Labcenter, Modena, Italy). To quantify viable cells numbers, PDOs were collected after 30 and 60 days and dissociated by means of a solution containing PBS 1X (Carlo Erba reagents, Cornaredo, Italy), DNase (Roche, Basilea, Switzerland) and Collagenase/Dispase (Roche, Basilea, Switzerland) enzymes. Cell viability and number was evaluated with Trypan Blue (Invitrogen, California, USA) and Countess Automated Cell Counter (Invitrogen, California, USA). Two different tests of cryoconservation and expansion were performed on PDOs grown for 3 weeks. *i*. PDOs were dissociated to obtain single-cell suspension; cells were resuspended in cryoprotective medium (12-132A, Lonza, Walkersville, MD, USA), frozen and maintained in the vapor phase of liquid nitrogen. After 1 month, cells were thawed, resuspended in fresh organoids’ medium, tested for their viability with Trypan Blue (Invitrogen, California, USA) and directly embedded in Matrigel to re-establish the organoids, according to protocols previously described. *ii.* whole PDOs were frozen in 50% conditioned medium and 50% cryoprotective medium (12-132A, Lonza, Walkersville, MD, USA). Organoids were maintained for 1 month in the vapor phase of liquid nitrogen, then thawed and cultured in organoids’ complete medium. Graphs and statistical analyses were performed using GraphPad Prism software (GraphPad, La Jolla, CA, USA). All data in graphs represent the mean of at least three independent experiments ± SEM.

### Histology

Immunohistochemical staining of PDOs was performed on 5 μm formalin fixed, paraffin-embedded tissue sections using a fully automated system (Bond-maX, Leica, Newcastle Upon Tyne, UK). Sections were de-waxed, rehydrated and incubated in retrieval buffer solution (Leica, Newcastle Upon Tyne, UK) for antigen recovery. Specimens were then washed with phosphate-buffered saline (pH 7.0) and incubated with the Bond Polymer Refine Detection Kit (Leica, Newcastle Upon Tyne, UK) according to the manufacturer’s protocols. Staining of proteins was performed with 3,3′-diaminobenzidine (DAB), and the slides were counterstained with Mayer’s haematoxylin (Diapath, Martinengo, Italy). Immunostains for NB84a (Leica; Newcastle, Clone NB84a, dilution 1:50), Synaptophysin (Dako, Clone DAK-SYNAP; dilution 1:100), Chromogranin A (Dako, Clone DAK-A3 dilution 1:200) and pHH3 (Thermo Scientific, dilution 1:100) were performed using an automated immunostainer. Images were acquired using Leica DM4000B microscope with Leica DFC295 camera. Measurement of the mitotic index was performed as follows: the percentage of positive tumour cell nuclei was counted in 5 × 100 cells for each case (magnification 400x, field size 0.18 mm^2^) in areas that are defined as “hot spots” (exactly where there are more positive nuclei).

### Array comparative genomic hybridisation (aCGH) analysis

aCGH was performed according to Agilent oligonucleotide array-based CGH for Genomic DNA Analysis Protocol (version 7.3). Samples were compared with commercial genomic DNA (Promega, Madison, Wisconsin, USA) representative for female and male. 1200 ng/μl of DNA were digested at high temperature and labelled by random priming with CY5-dCTP for the patients and CY3-dCTP for controls. Purification of the samples was performed by means of Micron filters YM-30 (Sigma-Aldrich, Missouri, USA) DNA was hybridised to a 244 K platform, at 65 °C for about 40 h. Chips were scanned on DNA microarray Agilent Scanner and digital analysis was carried out with Agilent Genomic Workbench 7.0 using the Aberration Algorithm ADM-1 with an Aberration Threshold of 5.0.

### Protein extraction and western blot analysis

PDOs were collected and dissociated in cold lysis buffer (Biosource International, USA) supplemented with Phosphatase Inhibitor Cocktail 2 (P5726-Sigma Aldrich, Missouri, USA), Phosphatase Inhibitor Cocktail 3 (P044-Sigma Aldrich, Missouri, USA), Protease Inhibitor Cocktail (P8340-Sigma-Aldrich, Missouri, USA) and PMSF (Sigma-Aldrich, Missouri, USA). Protein concentration was determined using Pierce BCA Protein assay kit (ThermoFisher Scientific, USA) and Wallac Victor spectrophotometer (Perkin Elmer, Massachusetts, USA) and loaded in Criterion TGX Precast gel (BioRad, California, USA). Membranes were incubated overnight at 4 °C with the following primary antibodies: anti-DBH (CSB-PA958833, Flarebio biotech LLC., MD, USA); anti-LGR-5 (CSB-PA267899, Flarebio biotech LLC., MD, USA); anti-MycN (CSB-PA965036, Flarebio biotech LLC., MD, USA); anti-p75 NGF receptor (ab93934, Clone 2F1C2, Abcam, Cambridge, UK); anti-Vimentin (NB100–74564, Clone J144, Novusbiologicals, Colorado, USA); anti-GAPDH (NB300–221, Clone 1D4, Novusbiologicals, Colorado, USA); anti-YAP (#4912, Cell Signaling, Massachusetts, USA). Images were acquired using Alliance 9.7 Western Blot Imaging system (Uvitec limited).

### In vitro limiting dilution colony assay

PDOs were collected and dissociated to obtain single-cell suspension as described above. Cells were plated into 96-well plates with decreasing numbers of cells per well (1000, 750, 500, 250, 125, 100, 50, 25, 10, 5, 2 and 1). Every dilution was replicated for 24 wells for three independent experiments. Cultures were monitored at light microscope for colony formation and, after 15 days of culture, wells were scored positive or negative for the presence of at least one colony. Data were analysed using extreme limiting dilution assay. Extreme limiting dilution analysis was performed using available software (http://bioinf.wehi.edu.au/software/elda/).

### Flow cytometry

PDOs were dissociated to obtain single-cells as described above. 200.000 cells/tube were stained for 30′ at room temperature in the dark with: anti-CD15 FITC (IM1423U, Clone 80H5, Beckman Coulter, California, USA), anti-CD44 PE (550,989, Clone 515, Becton Dickinson, California, USA), anti-CD133 PE (130-1,/1, Miltenyi Biotech, Cologne, Germany), anti-CD29 PE (556,049, Clone HUTS-21, Becton Dickinson, California, USA), anti-CD24 ECD (IM2645, Clone ALB9, Beckman Coulter, California, USA) and anti-CD56 PeCy7 (A21692, Clone N901, Beckman Coulter, California, USA). Samples were analyzed on FC500 flow cytometer (Beckman Coulter, USA): percentages of different populations were calculated based on live-gated cells relative to physical parameters, side scatter and forward scatter. At least 30.000 live gate events were acquired. Analyses were performed using Kaluza Flow Cytometry analysis software (Beckman Coulter, USA). Five independent experiments were performed.

## Results

### Establishment of NB-PDOs

**Fig. 1:**
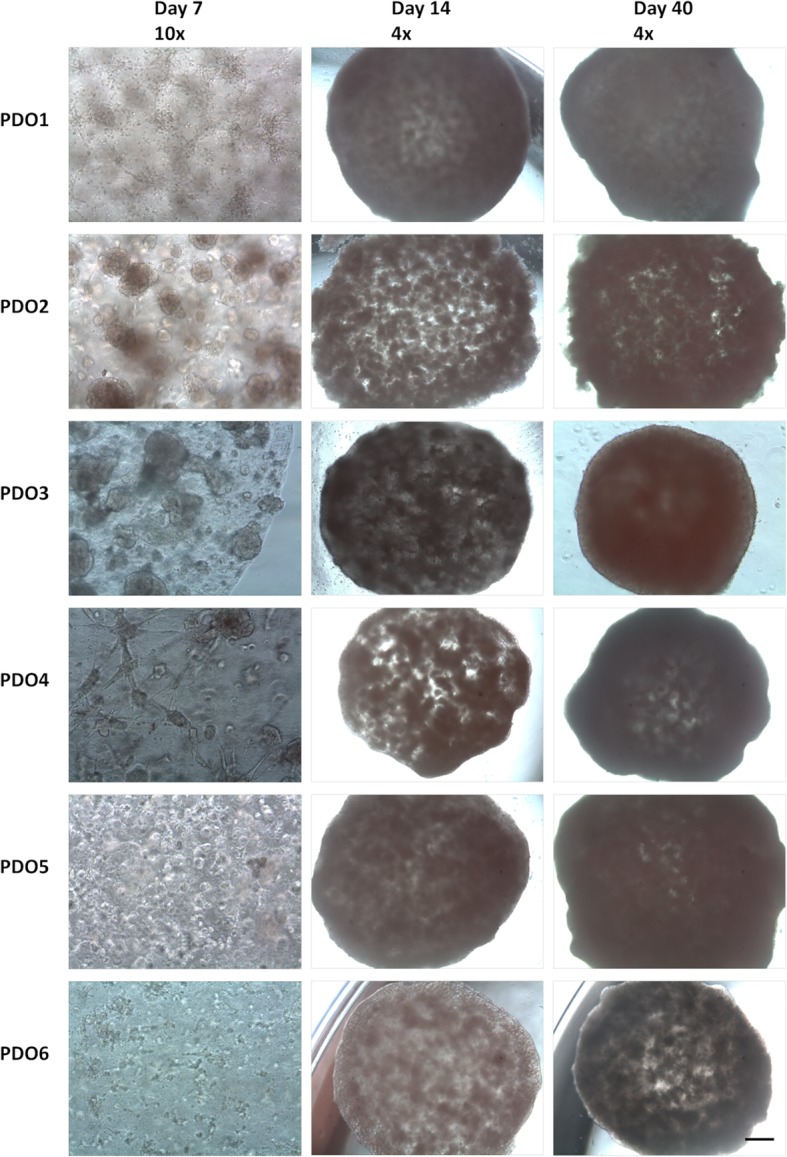
Establishment of NB-PDOs. Bright-field images of PDOs morphological changes on day 7, day 14 and day 40. The isolated cells formed compact 3D structure (left panel, magnification 10x, scale bar 100 μm) and PDOs increase in size along with the cultivation time (middle and right panel, magnification 4x, scale bar 600 μm)

We generated 6 independent NB organoids (PDO1-PDO6) corresponding to 4 different HR-NB patients stage M (Additional file 1: Table S1). The number of cells for PDOs, culture conditions and the time of culture were established for PDOs. PDOs were generated starting from 5 ×10^4^, 7.5×10^4^ and 1×10^5^ cells embedded in 20 μl Matrigel and cultured for a maximum of 2 months without passaging. We observed an efficient cell growth at all cellular concentrations tested, and we chose 1 × 10^5^ cells for the following experiments of the study. The formation and growth of PDOs was again monitored for 2 months. The morphology on day 7, 14 and 40 is shown in Fig. 1. The isolated cells are self-organized in a three-dimensional structure in which both spheres and single-cells are present (Fig. 1, left panels). As cells populate the Matrigel matrix, the organoids grow with cultivation time. To quantify viable cell number, a trypan blue assay was performed after 30 days and 60 days of culturing. Our data showed an increase in viable cell numbers within day 30 of culturing in all PDOs analysed (PDO1: 68.0% ± 7.1; PDO2: 79.4% ± 0.9; PDO3: 72.1% ± 2.2; PDO4: 72.6% ± 10.0; PDO5s; 85.4% ± 1.5; PDO6: 69.0% ± 6.6) (Additional file 3**:** Figure S1a). Although growth rates slowed in 5/6 PDOs (PDO1: 69.9% ± 11.8; PDO2: 77.7% ± 6.7; PDO3: 59.1% ± 20.7; PDO4: 87.8% ± 1.1; PDO5: 87.3% ± 1.1; PDO6: 76.6% ± 7.3), all PDOs were stable and viable after 2 months of continuous culture without passaging. To further characterise the proliferative feature of the PDOs, we evaluated the expression of the cell cycle markers phospho-Histone H3 (pHH3) by immunohistochemistry (Additional file 3: Figure S1b). Furthermore, to strengthen the findings on PDOs ability to organise themselves as parental tumours, we compared PDOs structure versus cells growing as spheres. We used AMC691B cells for the analysis since they have a high sphere-forming capacity. Although both PDOs and spheroids well reproduced NB’s cytology, spheroids did not resemble tumours structure, while in PDOs the architecture was well recognised and mimicking NB morphology (Additional file 4: Figure S2a). This morphology had the typical appearance observed in surgical specimens from NB patients. The analysis of the cell cycle markers pHH3 by immunohistochemistry was also performed on sphere samples (Additional file 4: Figure S2b). pHH3 expression revealed a comparable mitotic index of PDOs with NB tumours compared to spheroid culture.

In order to investigate the possibility of expanding and maintaining the organoids as reservoirs of patient-derived materials, we performed tests to evaluate the ability of PDOs to be cryopreserved and expanded. PDOs were dissociated obtaining single-cell suspension and cryopreserved. After 1 month, cells were thawed, tested for their viability (PDO1: 72% ± 5; PDO2: 79% ± 2; PDO3: 61% ± 5) and directly embedded in Matrigel to re-establish the PDO (Additional file 5: Figure S3a). In addition, whole organoids were also cryopreserved and thawed after 1 month (PDO1: 50% ± 3; PDO2: 63% ± 4; PDO3: 49.5% ± 6.5) (Additional file 5: Figure S3b). In both cases, the frozen-thawed PDOs were able to grow, indicating that cryopreservation did not affect the ability to proliferate in vitro, allowing their long-term storage and retrieval.

### PDOs recapitulate histological NB tumour characteristics

**Fig. 2:**
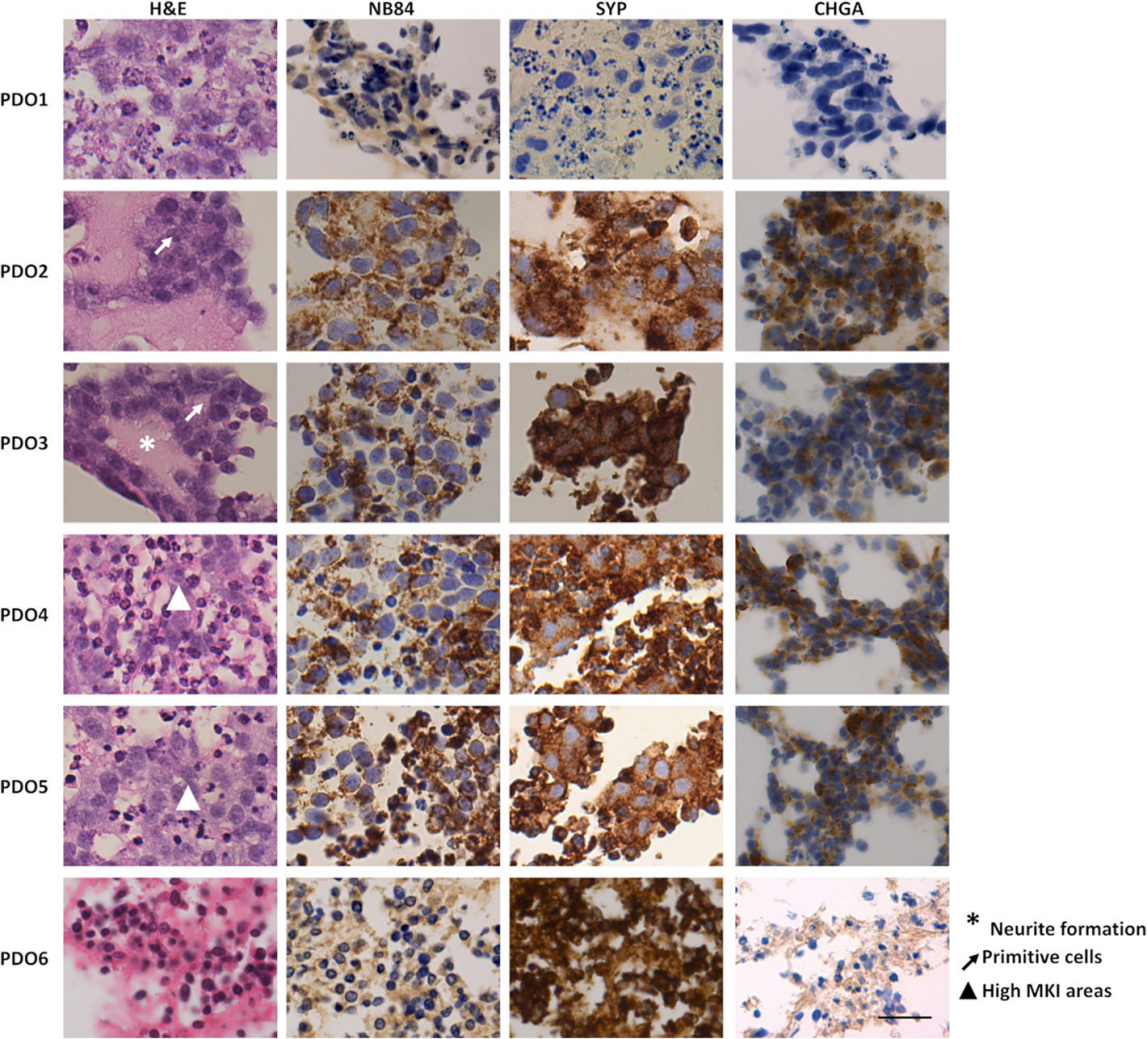
Histological analysis of NB-PDOs. Histological examination was performed on 5 μm formalin fixed, paraffin-embedded PDO sections. Left panels, H&E staining. Middle panels, NB84a and SYP staining. Right panels, CHGA staining. Scale bar 50 μm, magnification 40x. Asterisks: Neurite Formation; Arrows: Primitive cells; Arrowhead: MKI areas

After 40 days of culture, each PDO was paraffin-embedded, and paraffin blocks were cut to obtain 5 μm slices using a microtome. Haematoxylin-eosin (H&E) staining was performed in order to histologically characterise the PDOs in accordance with the procedure performed on the tumours of origin to classify its subtype. All primary tumours from which PDOs derived were classified as NB Schwannian stroma-poor, according to the International Neuroblastoma Pathology Committee (INPC). Moreover, at the diagnosis all tumours resulted positive for the NB markers NB84, SYP and CHGA. Histological images from primary tumour tissues can be found in the literature [10]. Similarly, all PDOs were correctly classified as Neuroblastoma, Schwannian stroma poor [3]. H&E staining highlighted the typical appearance of NB, characterised by uniformly sized cells, containing round to oval hyperchromatic nuclei and scant cytoplasm (Fig. 2, left panels). In all cases, histological examination showed a proliferation of primitive cells (arrows) with one or few prominent nucleoli, sometimes with recognisable neurite formation (asterisks) and variable mitotic and karyorrhectic activities (MKI areas, arrowhead). To further characterise the PDOs, we evaluated the expression of NB specific markers by immunohistochemistry. NB84 is a commonly employed diagnostic marker, and it was largely expressed in all the organoids examined, as evidenced by the brown cytoplasmatic staining (Fig. 2, middle panels). SYP is a transmembrane glycoprotein, also evaluated at diagnosis, and usually located at presynaptic vesicles of neurons and in vesicles in adrenal medulla. All PDOs resulted positive for this protein, depicted by the intense brown staining at the cytoplasm (Fig. 2, middle panels). CHGA is found in secretory vesicles of neurons and endocrine cells, but also widely expressed among NB tumours (Fig. 2, right panels). The positive staining for these specific markers confirmed that PDOs retained histological NB tumours features.

### PDOs exhibit NB specific chromosomal aberrations

**Fig. 3:**
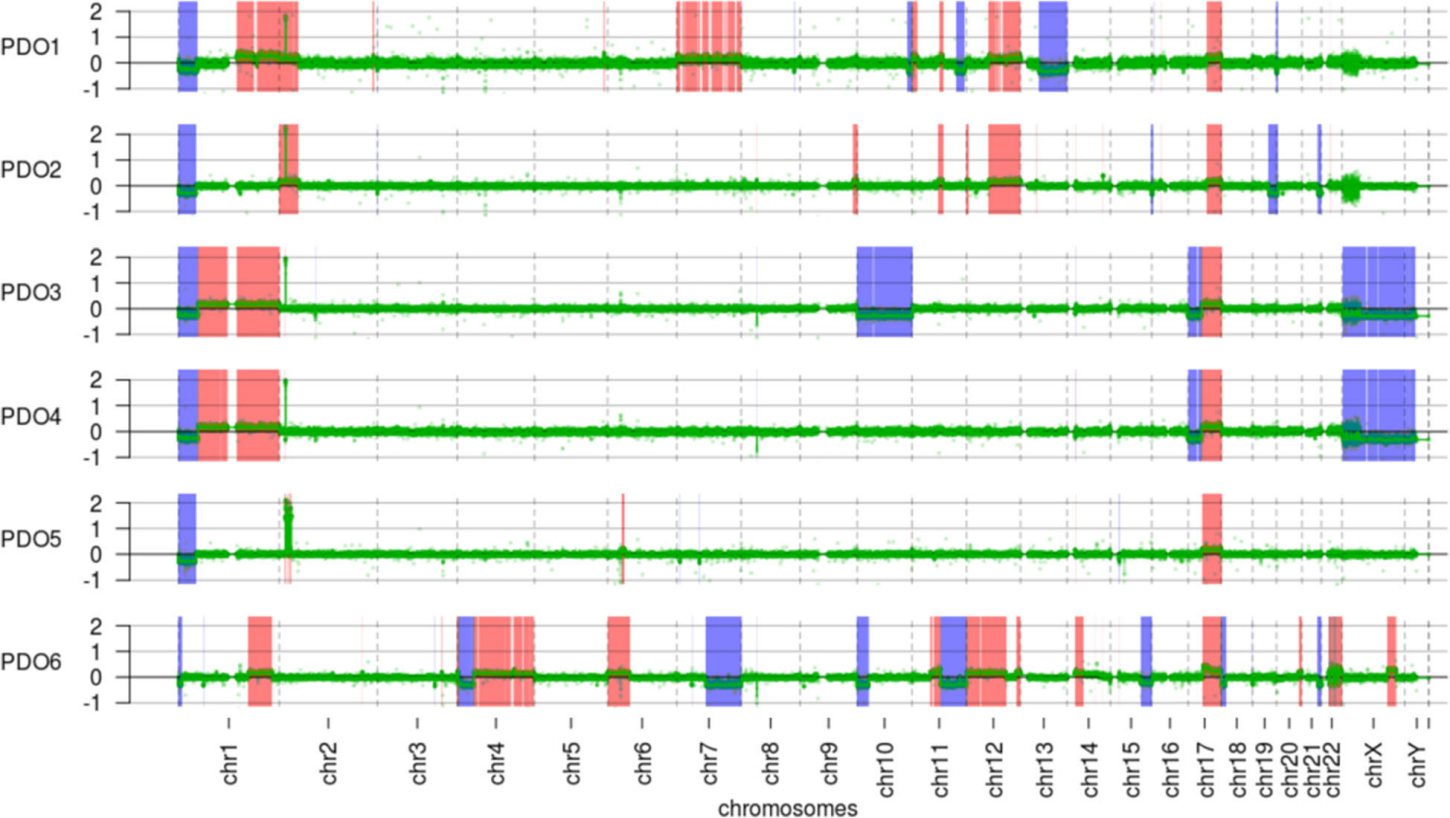
PDOs aCGH profiles. For each organoid, the x-axis of the graph represents the number of each chromosome, while the y-axis represents the extent of gain (red), loss (violet) or amplification (green peak) in logarithmic scale

To identify chromosomal abnormalities and to explore the genomic features of PDOs, we performed an array comparative genomic hybridization analysis (aCGH) on NB organoids. The genomic profiles of the PDOs recapitulated the majority of the common genomic alterations in NB: *MYCN* amplification, 1p deletion, 11q loss and 17q gain. We confirmed *MYCN* amplification, a NB hallmark related to poor outcome of the HR patients in patients older than 1 year [12], 1p deletion, 11q loss and 17q gain, that occurs in about 50% of the tumours [13]. These loci are linked to the aggressiveness of this tumour [14–17]. The PDOs aCGH profiles have been compared to the aCGH profiles of the tumours from which they derived, and the principal alterations are reported in Additional file 2: Table S2. Comparison between organoids and corresponding parental tumours showed that both their profiles are highly concordant (Fig. 3). aCGH profiles of the tumours of origin have been previously published [10]. Five out of six PDOs (PDO1, PDO2, PDO3, PDO4, PDO5) harbour *MYCN* amplification (green peaks in Fig. 3, Chr.2 p24.3), in accordance with the pattern observed in the original tumours. Furthermore, we detected 1p36 loss in 5/6 PDOs and the loss of the long arm of chromosome 11 only in 1/6 PDOs (violet peaks in Fig. 3). 17q gain was found in all the PDOs, whereas it was identified in 3/4 parental tumours (red peaks in Fig. 3). These apparently discordant data can be due to the presence of different sub-clones within primary NB tumours.

### PDOs retain stemness properties and heterogeneous cellular composition of NB tumours

**Fig. 4:**
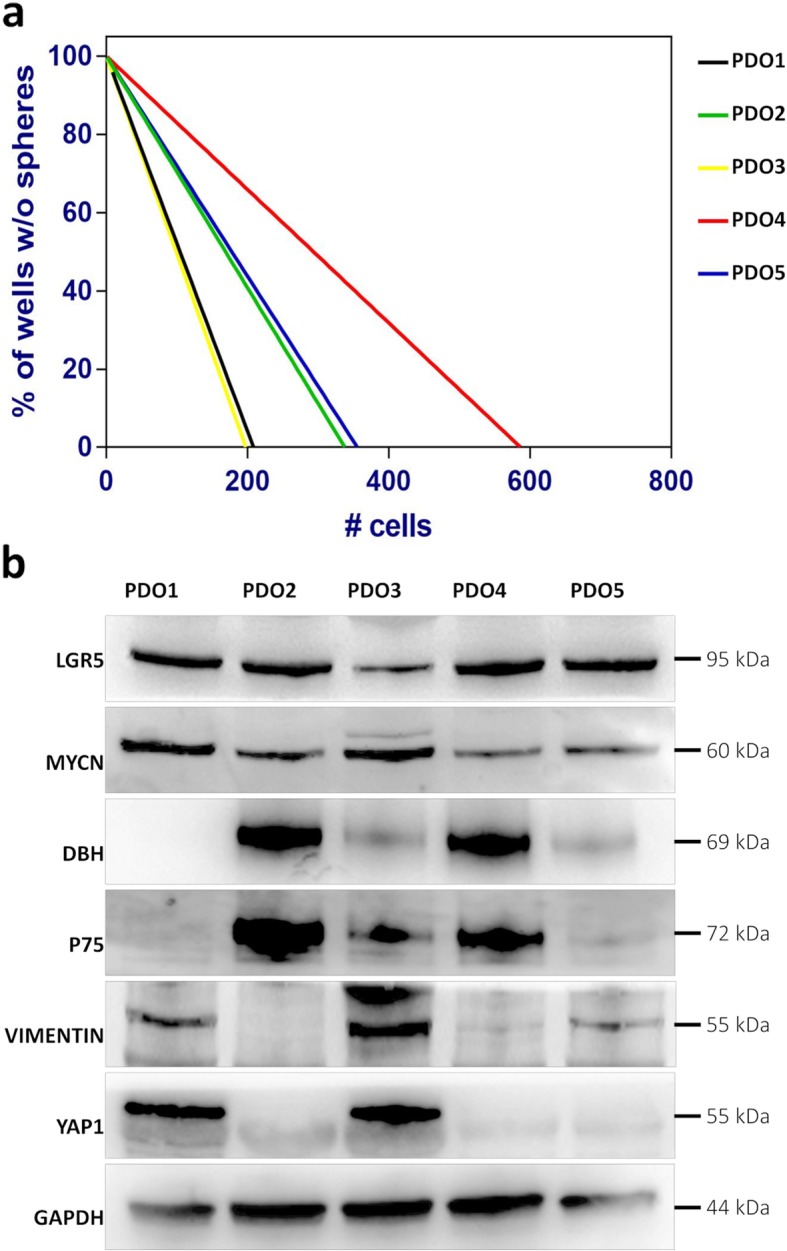
**a** Limiting dilution assay of PDOs. The graph represents the percentage of wells without spheres as a function of the number of cells. **b** Western blot analysis of adrenergic (DBH, p75) and mesenchymal (VIMENTIN, YAP1) markers and stemness marker (LGR5). We confirmed the protein expression of MYCN in all PDOs. The grouping of blots is cropped from different parts of the same gel. The images are representative of three independent experiments

We explored the molecular features of the PDOs model testing their functional properties by quantifying the stem cell population and tumour-initiating capability in vitro. After 2 months of culture, we performed limiting dilution assay (LDA) of 5 of 6 PDOs. Sphere-forming cell frequency calculated using ELDA software was as following: PDO1: 1/133; PDO2: 1/209; PDO3: 1/121; PDO4: 1/369; PDO5: 1/225 (Fig. 4a). We obtained different values of stem cell frequency that reflected the different ability to invade the Matrigel and fill its boundaries. Interestingly, PDOs that displayed a higher stem cell frequency (PDO1 and PDO3) were also able to populate earlier Matrigel droplet (Fig. 1).

We performed western blot analysis of 5 out of 6 PDOs. First, we examined LGR-5 marker since it is correlated to the stemness and it was recently presented as a cancer stem cell marker for NB cells [18]. We observed the cancer stem cell marker LGR-5 expressed in all PDOs. Next, we confirmed the protein expression of MYCN in all PDOs (Fig. 4b).

**Fig. 5:**
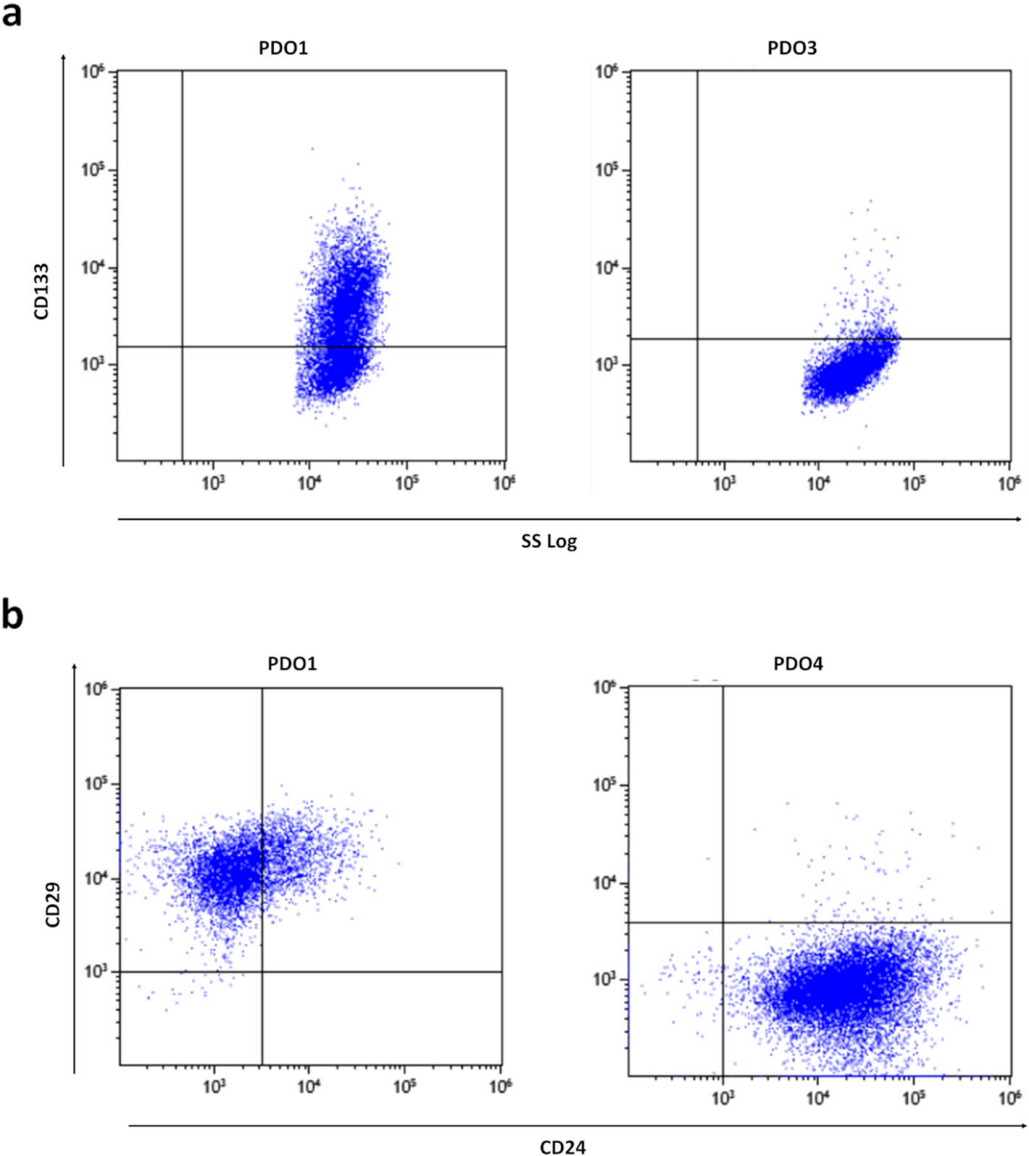
Flow cytometry analysis. Flow cytometry analysis of CD133 surface marker (**a**) and CD29 versus CD24 surface markers (**b**) on PDOs. Plots on the left are representative of PDO1, plots on the right are representative of PDO3 and 4 respectively, resembling PDO2–5. The plots are representative of more than three independent experiments

NB tumours includes two types of tumour cells: undifferentiated mesenchymal-like cells and committed adrenergic tumour cells, thus resembling cells from subsequent developmental stages of the adrenergic lineage [19]. Therefore, to explore mesenchymal and adrenergic features within the PDOs, we performed western blot analyses for the expression of the sympatho-adrenal lineage markers p75 (CD271) and dopamine beta-hydroxylase (DBH), and the mesenchymal markers VIMENTIN and YAP1. The analysis of these markers revealed two general categories: PDO2 and PDO4 displayed expression of adrenergic markers DBH and p75 and were negative for the mesenchymal markers VIMENTIN and YAP1; PDO1 and PDO3 were positive for VIMENTIN and YAP1, with low protein level of the adrenergic marker DBH and p75 (Fig. 4b). Interestingly, we evidenced that PDOs enriched in cells expressing VIMENTIN and YAP1 at higher levels have the highest stem cell frequencies, as highlighted by the LDA assay (graph in Fig. 4a, black and yellow lines). To further characterise the cell subtypes we performed flow cytometry analysis. In particular, we evaluated the expression of CD56 (NCAM), a neural cell adhesion molecule, CD133, a well-known neural stem cells marker [20], CD17, a type III receptor tyrosine kinase operating in cell signal transduction, CD15 and CD29 as neural stem cells markers, and CD24 as marker of differentiated cells and neuroblasts [21]. All PDOs except PDO1, characterised by a lower percentage, showed high positivity to CD56 marker, with more than 90% of positive cells (Table 1). Consistent with the observation that a subset of NB tumours with a favourable outcome is associated with CD117 (c-kit) expression [22], we investigated the expression pattern of this molecule. All PDOs resulted nearly negative for CD117, ranging from 0.3 to 9.5% of positive cells (Table 1). PDOs showed varying levels of expression of CD133. CD133 has been described to be associated with a mesenchymal phenotype [19]. Interestingly, CD133 resulted highly expressed in PDO1 (CD133+ cells: 58,4%) (Fig. 5a, left panel), the NB organoid we previously described as a mesenchymal subtype. Pruszak et al. [21] reported the identification, based on different expression of CD15, CD29 and CD24, of three distinct subpopulations of neural lineage cells derived from human embryonic stem cells: neural stem cells (CCD15+/CD29^HI^/CD24^LO^), neural crest-like/mesenchymal cells (CD15−/CCD29^HI^/CD24^LO^) and neuroblasts (CD15−/CD29^LO^/CD24^HI^). The PDOs we have analysed presented remarkable percentages of CD15−/CD29^LO^/CD24^HI^ cells, with PDO5 showing the highest proportion of neuroblasts (91.47%), followed by PDO2, PDO3, and PDO4 ranging from 70.8 to 77.6% (Fig. 5b, left panel). The lowest proportion was instead observed in PDO1, which is indeed characterised by the highest expression of the neural stem cell marker CD133 (Fig. 5b, right panel). This result again correlates with the observation that PDO1 is classified as undifferentiated mesenchymal subtype with respect to PDO2 and PDO4, characterised by a committed adrenergic phenotype. Lastly, to exclude the contamination of haematopoietic lineage cells, specific markers were investigated: CD14, CD45 and CD20. All PDOs resulted negative for these markers (Table 1).

**Table 1:** Table 1

	PDO1 mean %	PDO2 mean %	PDO3 mean %	PDO4 mean %	PDO5 mean %
CD56 (NCAM)	14.6	92.48	93.50	92.97	94.60
CD133	58.4	1.70	3.60	2.4	2.67
CD117 (c-kit)	6.1	0.50	6.17	9.5	0.30
CD24	10.7	93.74	93.48	93.35	96.78
CD15-CD29-CD24+ (ND)	0.04	70.81	72.16	77.63	91.47
CD14	0.3	1.2	0.6	0	0
CD20	1.2	0.3	1.35	0.75	0.95
CD45	2.4	1.65	2.85	1.70	0.80

Data are presented as mean percentage of *N* = 3 independent experiments

## Discussion

One of the major challenges for oncologists treating HR-NB is the high percentage of patients showing a fatal course with rapid disease progression despite multimodal therapy [4]. Next generation tools to recapitulate molecular and phenotypic landscape of the original NB tumour and to test personalised treatments are needed. Recently, the introduction of PDXs model for NB [23] and other tumours, including non-small cell lung cancer, malignant melanoma, breast cancer and others [9] [8], has helped the development of personalised cancer medicines by recapitulating the molecular, genotypic and phenotypic hallmarks of the patients’ tumours. As successfully demonstrated by Braekeveldt et colleagues [23], NB patient-derived orthotopic xenografts (PDOXs) retained the chromosomal copy number aberrations (1p del, *MYCN* amplification and 17q gain) and protein markers (SYP, p75, and tyrosine hydroxylase). However, although PDOXs are a promising model, their key limitations concern the abnormal immune system and tumour microenvironment of mice, and the inability to reproduce intra-tumour heterogeneity [24].

Recently, organoids technology obtained from healthy or diseased human tissue, has been used with great potential in multiple biomedical applications including disease modelling, drug screening, translational medicine, regenerative therapy and personalised therapy, and for biobanks [25]. To date, organoids have been established for multiple organs including liver [26] [27], small intestine [28] [29] [30], brain [31], and prostate [32] [33]. In NB, organoids have been established from commercial cell lines cultured in a microgravity rotary bioreactor that spontaneously aggregate into tumour-like structures [34, 35]. For the first time, we successfully established 6 organoids culture derived from primary tumour biopsies of HR-NB patients stage M that were maintained viable in culture up to 2 months and were able to reproduce tumour architecture and to preserve in vitro tumour heterogeneity. The use of an organoid model in NB allows us: i) to overcome the lack of experimental accessibility to in vivo systems; ii) to reproduce the architecture, heterogeneity and the complex nature of the biological processes of tumours of origin; iii) to overcome the current limitations of cell and animal models for pharmacological testing. Similarly, to NB-PDX model [23], we demonstrated that NB-PDOs retained the histological, cellular, molecular and genomic features of the NB of origin. Moreover, PDOs retained growing potential after both whole and single-cells cryopreservation, allowing their long-term storage and retrieval. Cells obtained from dissociated PDOs were able to organise in a tissue-like architecture, once embedded in Matrigel, allowing us to obtain a virtually unlimited supply of patient-specific material. We showed that PDOs are different from traditional spheres culture. Indeed, our findings revealed that both PDOs and spheroids reproduced NB cytology. However, the latter are arranged in disorganised structure while PDOs showed an organised architecture reproducing NB morphology. Furthermore, pHH3 staining on PDOs highlighted a mitotic index similar to that observed in NB patients’ tumours. We confirmed the positivity for the NB marker CD56, expressed on the surface of all tumours of neuroectodermal origin and widely accepted as a tumour marker [36].

NB tumours are highly heterogeneous and two cell types with shared genetic defects but extremely divergent phenotypes are observed in primary cells derived from HR-NB patients [19]. According to this evidence, we observed adrenergic or mesenchymal phenotype in PDOs based on the protein expression of specific markers, DBH/p75 expressed from the cells of sympathoadrenal lineage, and VIMENTIN/YAP1 associated to the mesenchymal phenotype. Moreover, we functionally quantified the stem cells population, obtaining different values of stem cell frequency for all PDOs. We highlighted that PDOs enriched in cells expressing VIMENTIN and YAP1 at higher levels (Fig. 4b) were also the PDOs with the highest stem cell frequencies, as emerged by the LDA assay (Fig. 4a, black and yellow lines). Furthermore, the expression of LGR-5 protein, a specific NB marker for cancer stem cells [18], in all PDOs suggests the preservation of cancer stem cells subpopulation. We investigated another relevant surface antigen, the transmembrane glycoprotein CD133, that has been correlated to the stemness of NB cells [37] and has been described to be the main marker differentially expressed between mesenchymal and adrenergic cells [19]. In our system, about 60% of PDO1 cells, which we defined as a mesenchymal subtype, exhibited CD133 expression. This result correlates with the observation that only PDO1 showed a low percentage of CD15−/CD29^LO^/CD24^HI^ cells, representing the subpopulation differentiating toward neuroblasts [21]. PDO3 showed differences compared to PDO1, having a low protein level expression of the adrenergic marker DBH and p75, although displaying a mesenchymal phenotype according to western blot analysis. These differences were evidenced by cytofluorimetric analysis revealing a low expression of CD133 for PDO3. Likely, these findings could indicate coexistence of two phenotypes in PDO3. Moreover, interestingly we found low CD117 (c-kit) expression for all PDOs confirming that PDOs resembles to subset of NB patients with a poor outcome.

## Conclusions

We were able to generate an alternative NB preclinical model exhibiting the features of NB tumour of origin and self-renewal properties as well as recapitulating NB tissue heterogeneity. PDOs grew efficiently even after cryopreservation, providing a reservoir of NB patients’ biological samples. PDOs could be used to improve the understanding of NB biology. However, further studies and analyses are mandatory to assess the use of PDOs for drug testing and to use them to identify the best chemotherapy combination for each patient, ultimately bringing the promise of personalised medicine to reality.

## Supplementary information

**Additional file 1: Table S1.** Clinical features of NB patients.

**Additional file 2: Table S2.** Genomic features of NB patients.

**Additional file 3: Figure S1.** PDO proliferative features.

**Additional file 4: Figure S2.** Spheroid cells.

**Additional file 5: Figure S3.** PDO cryopreservation and expansion.

## Data Availability

The datasets generated and/or analysed during the current study are available from the corresponding author on reasonable request.

## Abbreviations

aCGH: Array comparative Genomic Hybridisation
DBH: Dopamine beta-hydroxylase
H&E: Haematoxylin-eosin
HR: High-risk
INPC: International Neuroblastoma Pathology Committee
LDA: limiting dilution assay
NB: Neuroblastoma
OS: Overall survival
PDOs: Patient-Derived Organoids
PDOXs: Patient-derived orthotopic xenografts
PDX: Patient-derived tumour xenografts
pHH3: Phospho-Histone H3
SYP: Synaptophysin
