# Supplementary material for: Patient-derived organoids (PDOs) as a novel in vitro model for neuroblastoma tumours
Source: BMC Cancer. 2019 Oct 21;19:970. doi: 10.1186/s12885-019-6149-4 (PMC6802324; doi:10.1186/s12885-019-6149-4)
Supplement: Supplementary file 1 — Additional file 1: Table S1. Clinical features of NB patients. [file 12885_2019_6149_MOESM1_ESM.docx]

| **Additional file 1 Table S1: Clinical features of NB patients** | | | |
| --- | --- | --- | --- |
| **Patients** | **Age at diagnosis (range in months)** | **Site** | **Organoid** |
| **N691** | 70-80 | Adrenal | PDO1 Primary tumor  PDO2 Bone marrow metastasis |
| **N700** | 110-120 | Adrenal | PDO3 Primary tumor  PDO4 Bone marrow metastasis |
| **N711** | 10-20 | Abdominal side chain | PDO5 Primary tumor |
| **N772** | 30-40 | Adrenal | PDO6 Primary tumor |
| Adapted from Bata-Eya et al, 2014. | | | |
