# Supplementary material for: Patient-derived organoids (PDOs) as a novel in vitro model for neuroblastoma tumours
Source: BMC Cancer. 2019 Oct 21;19:970. doi: 10.1186/s12885-019-6149-4 (PMC6802324; doi:10.1186/s12885-019-6149-4)
Supplement: Supplementary file 2 — Additional file 2: Table S2. Genomic features of NB patients. [file 12885_2019_6149_MOESM2_ESM.docx]

| **Additional file 2 Table S2: Genomic features of NB patients** | | | |  |
| --- | --- | --- | --- | --- |
| **Patients** | **1p** | **11q** | **17q** | **MYCN**  **Amplification** |
| **N691** | 1p36 Loss | Normal | 17q21 Gain | Yes |
| **N700** | 1p36 Loss | Normal | Normal | Yes |
| **N711** | 1p36 Loss | Normal | 17q21 Gain | Yes |
| **N772** | Normal | 11q13 Loss | 17q21 Gain | No |
| Adapted from Bata-Eya et al, 2014. | | | |  |
