# Supplementary figures and images for: Patient-derived organoids (PDOs) as a novel in vitro model for neuroblastoma tumours
Source: BMC Cancer. 2019 Oct 21;19:970. doi: 10.1186/s12885-019-6149-4 (PMC6802324; doi:10.1186/s12885-019-6149-4)

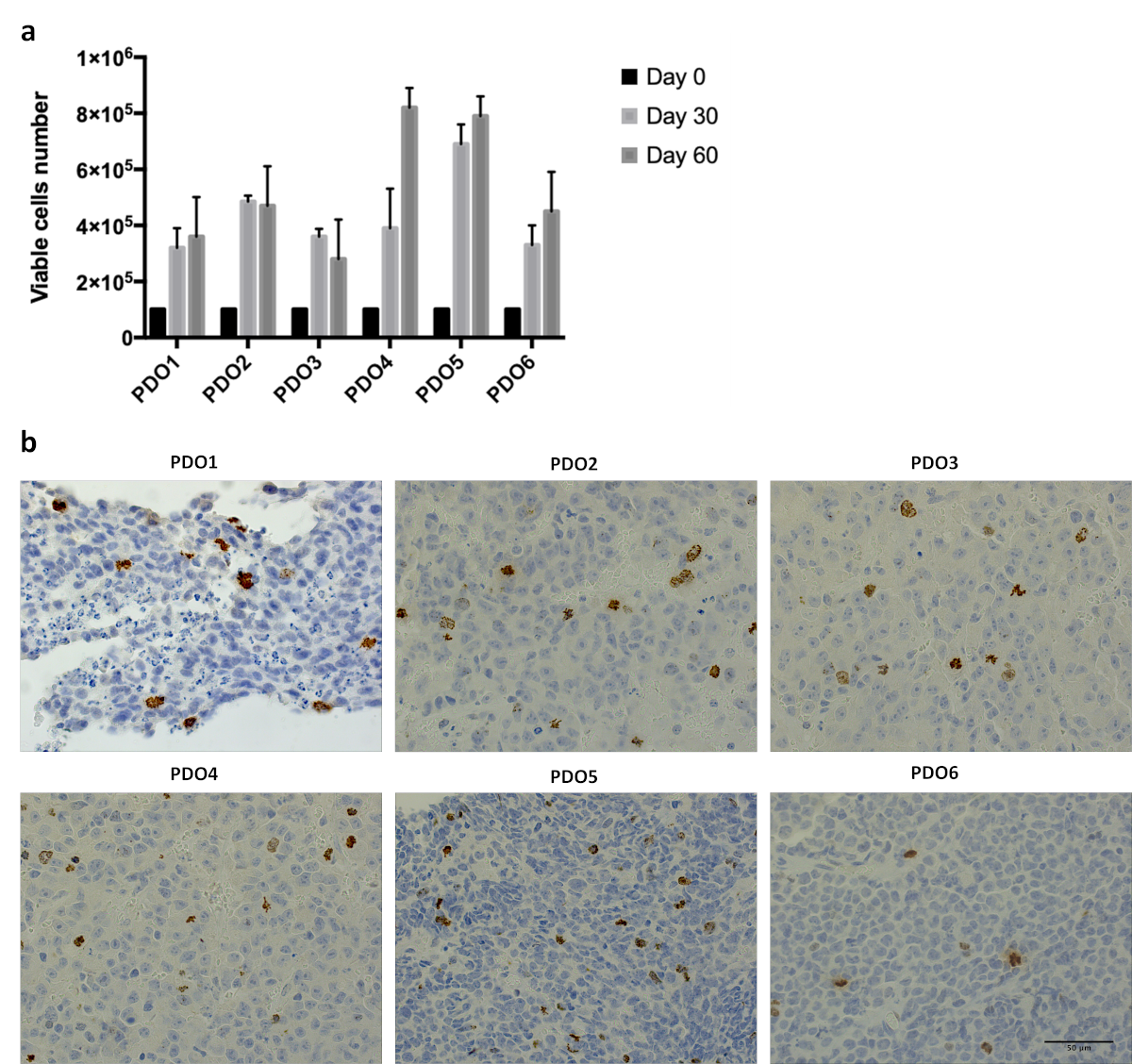

Supplement: Supplementary file 3 — Additional file 3: Figure S1. PDO proliferative features. [file 12885_2019_6149_MOESM3_ESM.png]

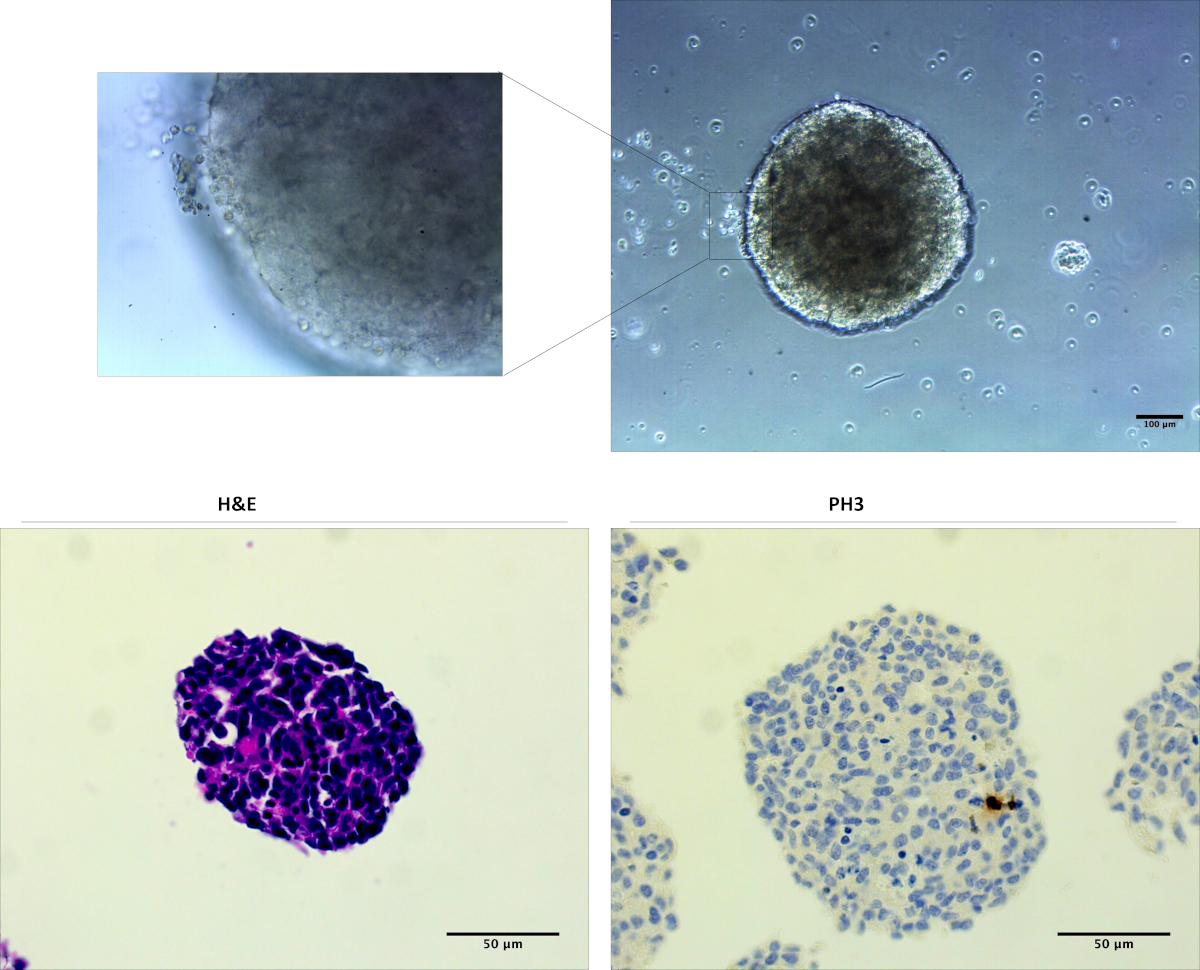

Supplement: Supplementary file 4 — Additional file 4: Figure S2. Spheroid cells. [file 12885_2019_6149_MOESM4_ESM.png]

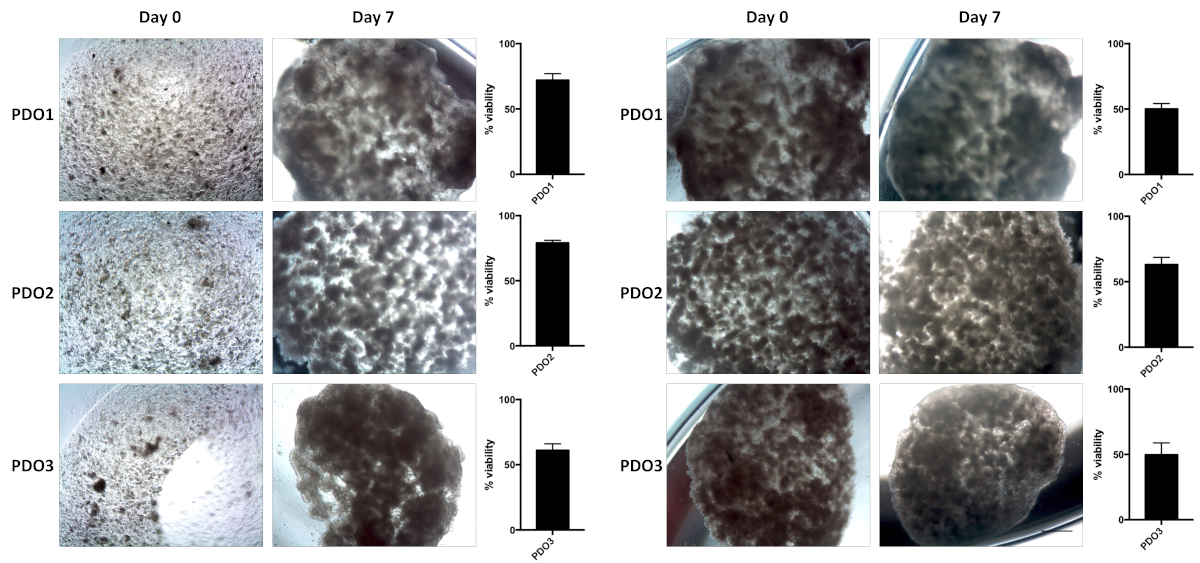

Supplement: Supplementary file 5 — Additional file 5: Figure S3. PDO cryopreservation and expansion. [file 12885_2019_6149_MOESM5_ESM.png]
